# Multi-modality imaging to assess metabolic response to dichloroacetate treatment in tumor models

**DOI:** 10.18632/oncotarget.13176

**Published:** 2016-11-07

**Authors:** Marie-Aline Neveu, Géraldine De Preter, Nicolas Joudiou, Anne Bol, Jeffery R. Brender, Keita Saito, Shun Kishimoto, Vincent Grégoire, Bénédicte F. Jordan, Murali C. Krishna, Olivier Feron, Bernard Gallez

**Affiliations:** ^1^Biomedical Magnetic Resonance Research Group, Louvain Drug Research Institute, Université catholique de Louvain, Brussels, Belgium; ^2^Radiation Oncology Department & Center for Molecular Imaging, Radiotherapy & Oncology, Institute of Experimental and Clinical Research, Université catholique de Louvain, Brussels, Belgium; ^3^Radiation Biology Branch, National Cancer Institute, NIH, Bethesda, USA; ^4^Pole of Pharmacology and Therapeutics, Institute of Experimental and Clinical Research, Université catholique de Louvain, Brussels, Belgium

**Keywords:** tumor metabolism, DCA, ^17^O MRS, hyperpolarized ^13^C-MRI, ^18^F-FDG PET

## Abstract

Reverting glycolytic metabolism is an attractive strategy for cancer therapy as upregulated glycolysis is a hallmark in various cancers. Dichloroacetate (DCA), long used to treat lactic acidosis in various pathologies, has emerged as a promising anti-cancer drug. By inhibiting the pyruvate dehydrogenase kinase, DCA reactivates the mitochondrial function and decreases the glycolytic flux in tumor cells resulting in cell cycle arrest and apoptosis. We recently documented that DCA was able to induce a metabolic switch preferentially in glycolytic cancer cells, leading to a more oxidative phenotype and decreasing proliferation, while oxidative cells remained less sensitive to DCA treatment. To evaluate the relevance of this observation in vivo, the aim of the present study was to characterize the effect of DCA in glycolytic MDA-MB-231 tumors and in oxidative SiHa tumors using advanced pharmacodynamic metabolic biomarkers. Oxygen consumption, studied by ^17^O magnetic resonance spectroscopy, glucose uptake, evaluated by ^18^F-FDG PET and pyruvate transformation into lactate, measured using hyperpolarized ^13^C-magnetic resonance spectroscopy, were monitored before and 24 hours after DCA treatment in tumor bearing mice. In both tumor models, no clear metabolic shift was observed. Surprisingly, all these imaging parameters concur to the conclusion that both glycolytic tumors and oxidative tumors presented a similar response to DCA. These results highlight a major discordance in metabolic cancer cell bioenergetics between in vitro and in vivo setups, indicating critical role of the local microenvironment in tumor metabolic behaviors.

## INTRODUCTION

Warburg metabolism (enhanced glycolysis in the presence of oxygen) is a common feature of several malignant tumors and is associated with cancer aggressiveness, invasiveness and poor prognosis [1–3]. Because of this high glycolytic rate in various cancers, targeting glucose metabolism is presented as an attractive anticancer approach endowed with a high specificity and limited undesirable side effects [4, 5]. Indeed, conventional treatments rely on the rapid proliferation process present in cancer cells but also in healthy cells. Treatments targeting glycolytic metabolism should instead specifically alter metabolic adaptations that support the Warburg malignant phenotype, adaptations that are not shared by normal cells. To support drug development and assessment in clinical trials, there is a critical need for dedicated criteria evaluating tumor response to these emerging therapies. Moreover, for new cytostatic agents targeting tumor metabolism, the use of conventional anatomical imaging techniques is not optimal for treatment response assessment [4] and only functional and molecular imaging techniques may offer the possibility of an early assessment of the tumor response [6–8].

Recently, we have investigated the effects of dichloroacetate (DCA) in tumor cell lines presenting different metabolic profiles [9]. DCA is a promising molecule that promotes glucose oxidation over glycolysis by inhibiting the mitochondrial pyruvate dehydrogenase kinase (PDK) and has successfully reached clinical trials [10]. We found that 5 mM DCA was more effective in glycolytic-phenotype cancer cells, where reduction in cell proliferation was mediated by a reactivation of mitochondrial function and a decrease in glycolytic and pentose phosphate pathway fluxes. Our data suggested that DCA may benefit to patients with highly glycolytic tumors. Therefore, the objective of the present study was to assess the effect of DCA in these prototypical tumor models in vivo, namely the glycolytic MDA-MB-231 human breast cancer model and the oxidative SiHa human cervical cancer model. For this purpose, we used a multi-modality molecular imaging approach using several pharmacodynamic metabolic biomarkers. Oxygen consumption, studied by ^17^O magnetic resonance spectroscopy (^17^O MRS), glucose uptake, evaluated by ^18^F-fluorodeoxyglucose positron emission tomography (^18^F-FDG PET) and pyruvate transformation into lactate, measured during hyperpolarized ^13^C-magnetic resonance imaging (hyperpolarized ^13^C-MRI), were monitored before and after DCA treatment in tumor bearing mice. Surprisingly, in vivo models did not recapitulate the previously observed in vitro behavior.

## RESULTS

**Figure 1:**
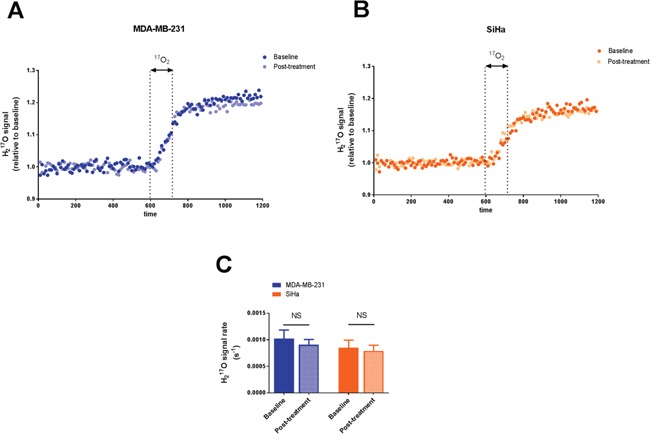
Effect of dichloroacetate on tumor oxygen consumption in vivo Tumor H_2_^17^O signal from representative MDA-MB-231 tumors **A.** and SiHa tumors **B.** acquired before, during and after a 2 min inhalation period of the ^17^O_2_ gas. H_2_^17^O signal is expressed as relative to the mean baseline signal before ^17^O_2_ delivery. ^17^O_2_ metabolismis not modified by DCA treatment. **C.** Comparison of the rate of H_2_^17^O signal after ^17^O_2_ delivery in tumors pre and post-treatment. Data are expressed as means ± SEM. Paired tests were two-sided.

**Figure 2:**
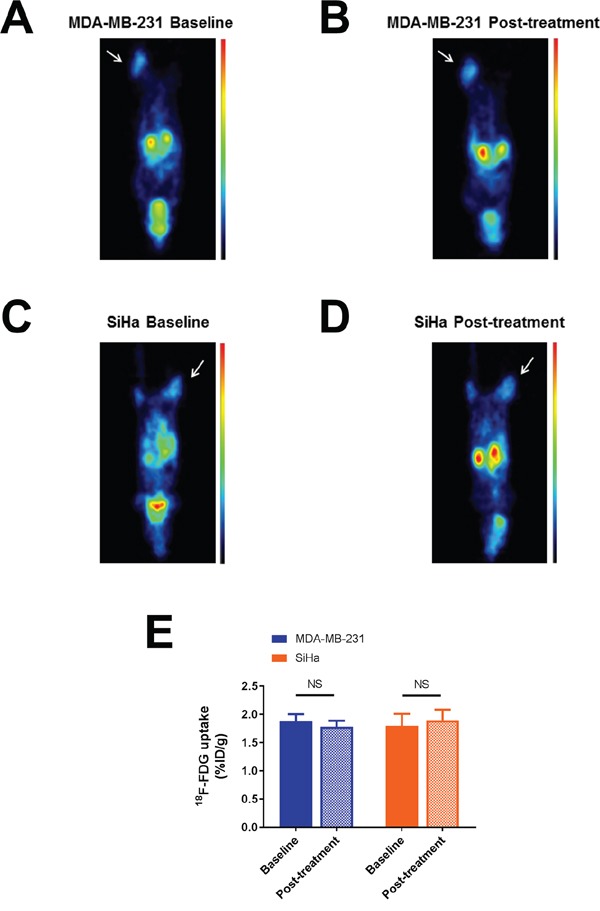
Effect of dichloroacetate on tumor glucose uptake in vivo Representative ^18^F-FDG PET images showing MDA-MB-231 **A-B.** and SiHa **C-D.** tumor-bearing mouse imaged before and 24 hours after DCA treatment. Tumors are indicated by thin arrows. ^18^F-FDG uptake is expressed in %ID/g. Images were normalized. DCA does not alter ^18^F-FDG uptake in MDA-MB-231 and SiHa tumors. **E.** Comparison of ^18^F-FDG uptake before and after treatment. Data are expressed as means ± SEM. Paired tests were two-sided.

**Figure 3:**
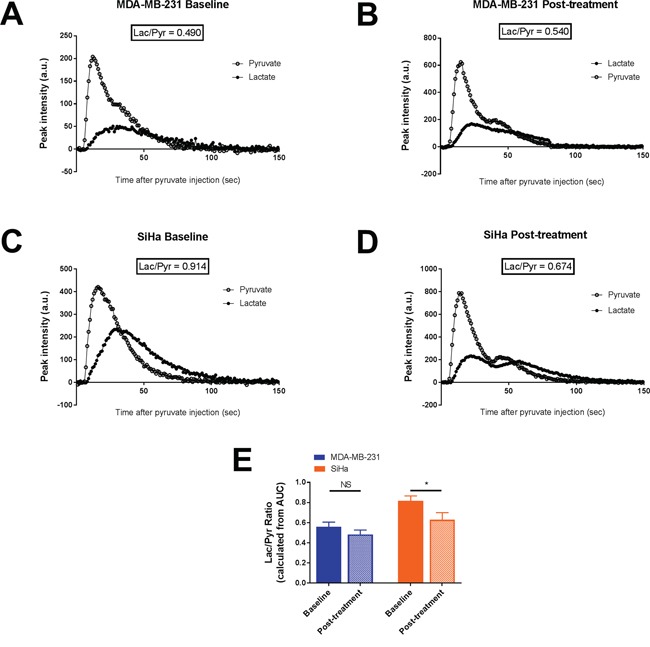
Effect of dichloroacetate on tumor lactate production in vivo Tumor lactate and pyruvate peak intensities after i.v. injection of hyperpolarized 1-^13^C pyruvate from representative MDA-MB-231 tumors **A-B.** and SiHa tumors **C-D.** Lactate production, measured by the Lac/Pyr ratio, in MDA-MB-231 and SiHa tumors before and after treatment **E.** Data are expressed as means ± SEM. Paired tests were two-sided.

To assess the impact of DCA treatment on the metabolism of the models in vivo, oxygen consumption (Figure 1), glucose uptake (Figure 2) and lactate flux (Figure 3) were measured in MDA-MB-231 and SiHa tumors before and 24 hours after DCA treatment.

In Figure 1, tumor H_2_^17^O spectra are presented for representative MDA-MB-231 (Figure 1A) and SiHa tumors (Figure 1B) during ^17^O MRS experiments. The results were highly reproducible under the same conditions tested. The evolution of H_2_^17^O signal, demonstrating the ^17^O_2_ metabolism in tumors, was similar before and after DCA treatment in MDA-MB-231 tumors (Figure 1A) and in SiHa tumors (Figure 1B). In both tumors models, we found that DCA treatment did not majorly impact oxygen consumption, as assessed by the rate of increase in H_2_^17^O signal (Figure 1C). MDA-MB-231 tumors exhibited a slope of 1.02 10^-3^ ± 0.16 10^-3^ s^-1^ and 0.91 10^-3^ ± 0.09 10^-3^ s^-1^ before and after treatment respectively (n=5, P=0.5366). For SiHa tumors, the slopes were 0.85 10^-3^ ± 0.14 10^-3^ s^-1^ under baseline condition and 0.79 10^-3^ ± 0.11 10^-3^ s^-1^ post-treatment (n =5, P=0.2892).

^18^F-FDG uptake (%ID/g) measured in both tumor models under baseline and post-treatment conditions are presented in Figure 2. In both tumor models, we found that DCA treatment did not lead to a significant change in the uptake of ^18^F-FDG (Figure 2A-2D), assessing a limited impact of DCA on glucose uptake (Figure 2E). %ID/g measured on PET images (mean ± SEM) were 1.88 ± 0.12 under baseline condition and 1.78 ± 0.11 after treatment for MDA-MB-231 tumors (n=7, P=0.2120) and 1.79 ± 0.21 under baseline condition and 1.89 ± 0.19 after treatment for SiHa tumors (n=7, P=0.0813).

The influence of DCA treatment on pyruvate transformation into lactate was measured after hyperpolarized 1-^13^C pyruvate injection during hyperpolarized ^13^C-MRS studies (Figure 3). Representative pyruvate and lactate peak intensities over time of MDA-MB-231 tumors and SiHa tumors captured before and 24 hours after DCA treatment are shown in Figure 3A-3D. Lactate production was reduced after DCA treatment only in SiHa tumors (Figure 3E). Lactate/pyruvate ratio (Lac/Pyr) shifted from 0.55 ± 0.05 to 0.48 ± 0.04 in MDA-MB-231 tumors (n=7, P=0.3105) and from 0.82 ± 0.05 to 0.63 ± 0.07 in SiHa tumors (n=7, P=0.0348).

**Figure 4:**
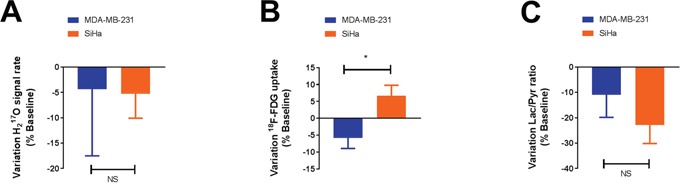
DCA does not significantly influence the metabolism of glycolytic tumors compared to oxidative tumors, as assessed by ^17^O2 metabolism A., ^18^F-FDG uptake B. and pyruvate transformation into lactate C. measurements in vivo The magnitude of response to dichloroacetate (variation) is identical in both models, only a small difference in behavior is observed for ^18^F-FDG uptake. Data are expressed as means ± SEM. Unpaired tests were two-sided.

We also evaluated the magnitude of response to the treatment by measuring the variation within the above biomarkers between baseline and post-treatment conditions (Figure 4). No differences in oxygen consumption and lactate flux measurements were observed between MDA-MB-231 and SiHa tumors during the treatment (P>0.05) (Figure 4A, 4C). Only a small but significant difference in behavior was observed for ^18^F-FDG uptake measurements (P=0.0240) (Figure 4B). MDA-MB-231 tumors decreased their ^18^F-FDG uptake after treatment (n=7, -5.4 ± 3.5 %) compared to SiHa tumors (n=7, +6.7 ± 3.1 %).

## DISCUSSION

In this study, the impact of DCA on tumors presenting different metabolic profiles was evaluated using molecular imaging in vivo. Recent findings identified that DCA preferentially impairs glycolytic cells compared to oxidative cells. The purpose of the present study was to establish the relevance of these findings in vivo using the same prototypical tumor models as in our in vitro study [9], namely the MDA-MB-231 human breast cancer model reported as glycolytic [1, 11] and the SiHa human cervical cancer model documented as oxidative [11, 12]. The dose and administration scheme were selected based on previous reports attesting the efficacy of DCA in tumors [13–15].

In vitro, we previously identified clear effects of DCA treatment on oxygen consumption, glucose consumption and lactate uptake in glycolytic MDA-MB-231 human breast cancer cells. On the other hand, the metabolic activity of oxidative SiHa human cervical cancer cells was not altered by DCA treatment. Using a multi-modality imaging project, we were not able to recapitulate these findings in vivo. Pre-treatment, MDA-MB-231 and SiHa tumors exhibit the same metabolic profile. As MDA-MB-231 and SiHa tumors were previously described as hypoxic under baseline condition [16], we highlighted here that both tumor models exhibit a glycolytic phenotype under anaerobic condition. Post-treatment, glycolytic MDA-MB-231 tumors do not appear more impacted than oxidative SiHa tumors (Figure 1-4). Also, some marginal metabolic changes were identified such as a significant decreased lactate production in SiHa tumors (Figure 3E) or a decreased ^18^F-FDG uptake in MDA-MB-231 tumors (Figure 4B). Together, those findings did not highlight a clear metabolic shift in MDA-MB-231 tumors or in SiHa tumors treated with DCA during 24 hours (Supplementary Figure S1). This inability to observe any treatment response in vivo could not be attributed to any differences in growth rate between the tumor models under study (Supplementary Figure S2). Also, the measurement repeatability was formerly established using the same tumor models [16]. Our study demonstrated that the tumor metabolic response to DCA was dramatically different between in vitro and in vivo conditions.

Because of its good tolerability and safety, DCA has been universally exploited to lower lactate levels in acquired or congenital forms of lactic acidosis [17]. In 2007, Bonnet and colleagues investigated the effects of DCA in cancer and discovered that DCA was promoting apoptosis in vitro and decreasing tumor growth in vivo [18]. Since then, this orally available and cheap molecule has been further investigated in vitro, in vivo and successfully reached clinical trials. The first data available from the clinical trials indicate that DCA appears to be efficient in adults in solid and brain tumors [19, 20]. However, no firm conclusions stand out in advanced non-small cell lung cancer [21]. In another recent study of Feuerecker and co-workers, promotion of tumor growth was even observed after DCA treatment in neuroblastoma tumors [22]. These studies indicate that response to DCA treatment may drastically vary among tumor types.

The redirection of glucose metabolism from glycolysis to oxidation leading to the inhibition of proliferation and the induction of caspase-mediated apoptosis was initially proposed as the generic mechanism of action of DCA. In a recent phase I study in patients with advanced solid tumors, decreased ^18^F-FDG uptake was observed after DCA therapy, supporting the use of ^18^F-FDG uptake as a potential biomarker of response to DCA [20]. Also, hyperpolarized ^13^C-pyruvate MRI has already been used in several preclinical studies to monitor DCA effect in solid tumors [13–15], but also in cardiac [23, 24] and brain studies [25, 26]. In the present study, ^18^F-FDG uptake was unchanged after DCA treatment (Figure 2E). This suggests that DCA treatment does not impair glucose uptake and phosphorylation but could potentially impact downstream transformation of glucose. However, no effects on bicarbonate production were detected that could demonstrate changes in energy metabolism from glycolysis to oxidative phosphorylation (Supplementary Figure S3). Recent findings suggested that DCA may also act by other mechanisms. While a possible disruption of the balance between fatty acid β-oxidation and glucose oxidation has already been suggested as an additional mechanism involved in the overall effects of DCA in vivo (as reviewed by [27]), PDK inhibitors may potentially induce other compensatory mechanisms that could limit the impact of such drugs on global tumor metabolism. The anti-cancer effects of DCA appear to rely on multiple mechanisms depending on the drug concentration, drug administration scheme [28] and cell type [29]. Also, a change in PDK isoform expression between in vitro and in vivo model could also greatly influence the effect of DCA on tumor metabolism in vivo. Indeed, oncogene regulation and tumor microenvironment, like extracellular acidosis, can affect PDK isoform expression [30], possibly leading to the expression of a PDK isoform less sensitive to DCA effects. Our findings are consistent with a recent study highlighting that tumor microenvironment could be as important as the (epi) genetic profile to shape the tumor phenotype [31]. Further investigations using relevant isogenic cell clones with ability to form tumors in vivo should be considered to determine the effects of DCA on energy metabolism in vivo.

In conclusion, our multi-modality imaging study identified major discordances between in vitro and in vivo metabolic responses to DCA treatment, in cancer models presenting distinct metabolic profiles. Results suggest preferring implanted tumors and spontaneous cancer models to study DCA treatment within the milieu of the tumor microenvironment. Overall, further investigations are required to elucidate the impact of different tumor microenvironments on metabolic effects of DCA and its impact for clinical use.

## MATERIALS AND METHODS

### Cell culture

MDA-MB-231 (human breast cancer) and SiHa (human cervix squamous cell carcinoma) cell lines (American Type Culture Collection [ATCC]), were routinely cultured in Dulbecco's modified Eagle's medium containing 4.5g/l glucose supplemented with 10% fetal bovine serum and 1% penicillin-streptomycin.

### Animal housing

Animal studies were undertaken in accordance with Belgian and the Université catholique de Louvain ethical committee regulations (agreements number UCL/2010/MD/001 and UCL/2014/MD/026). Hyperpolarized ^13^C-MRI experiments were carried out in compliance with the Guide for the Care and Use of Laboratory Animal Resources (National Research Council, 1996) and approved by the National Cancer Institute (NCI) Animal Care and Use Committee.

### Tumor implantation and animal experiments

A total of 10^7^ MDA-MB-231 cells or 10^7^ SiHa cells, amplified in vitro, were collected by trypsinization, washed three times with Hanks balanced salt solution and resuspended in 200 μL of a 1:1 mixture of Matrigel (BD Biosciences) and Hanks balanced salt solution. For ^17^O MRS and PET scan experiments, the tumor cells were inoculated subcutaneously into the hind thigh of nude NMRI female mice (Janvier Le Genest-Saint-Isle, France). For hyperpolarized ^13^C-MRI experiments, the tumor cells were inoculated subcutaneously into the hind thigh of athymic nude female mice (Frederick Cancer Research Center, Animal Production, Frederick, MD, USA). The experiments were performed when tumors reached 7 mm (at this tumor size, necrosis was less than 5% as characterized by Hematoxylin Eosin staining).

**Figure 5:**
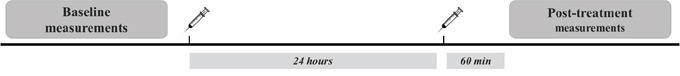
Experimental protocol

To assess the effects of DCA on tumor metabolism using biomarkers, all animals have undergone imaging before and after treatment, with one day between each measurement. Dichloroacetate sodium (Sigma-Aldrich) was administered intraperitoneally (200 mg/kg) after baseline measurements to the mouse. Another dose was given 24 hours after the first dose injection. Post-treatment measurements were initiated 1 hour after treatment administration. This administration scheme is consistent with previous studies attesting the effects of DCA in tumors using hyperpolarized ^13^C-MRI [13, 15]. The imaging protocol is summarized in Figure 5.

Mice were anesthetized by isoflurane inhalation (Forene, Abbot, England) mixed with air in a continuous flow (2 L/min). Animals were warmed (approximately 35°C) throughout the anesthesia period.

### ^17^O MRS experiments

For oxygen consumption experiments, ^17^O MRS was performed on an 11.7 T (Bruker, Biospec) controlled by Paravision 6.0 (Bruker, Ettlingen, Germany). Experiments were carried out using a ^1^H/^17^O Bruker surface coil system positioned over the tumor mass. Anatomical images were firstly acquired using a T_2_-weighted axial turbo RARE sequence (TR = 2500ms; TE = 30ms; rare factor = 8; NA = 2; FOV = 25x25mm^2^; resolution: 98 x 98 μm^2^; 1 mm slice thickness). ^17^O MRS measurements were carried out using a nonlocalized, single-pulse sequence (TR = 16.5 ms; NA = 600; repetition: 120; T_acq_ = 20 min; Acq BW = 5000 Hz; FA = 20°). For ^17^O MRS sequence, the 90° reference pulse was optimized previously on natural abundance H_2_^17^O samples.

To measure tumor oxygen consumption during the ^17^O_2_ delivery, a total of 120 ^17^O-spectra were collected in about 20 min, before, during and after a 2 min inhalation period of the ^17^O_2_ mixture. The integrals of the H_2_^17^O peaks over time were measured using a home-made program written in Matlab (The MathWorks Inc., Natick, MA, USA). H_2_^17^O signal was then expressed as relative to the mean baseline signal before ^17^O_2_ delivery. The mean signal of the final steady state (s_final_) during the post-inhalation period was calculated between 1100 and 1200 s. We considered that the steady state was reached when the signal stood between s_final_ ± 5 % of signal variation. The slope during the linear incorporation phase was measured between 600 sec and the time point when steady state was reached.

### PET/CT imaging

Whole-body PET imaging was performed on a dedicated small-animal PET scanner (Mosaic, Philips Medical Systems, Cleveland, USA) with a spatial resolution of 2.5 mm (FWHM). The PET scans were followed by whole-body acquisitions using a helical CT scanner (NanoSPECT/CT Small Animal Imager, Bioscan Inc., DC, USA). For each breathing condition, anesthetized mice were injected 120 μl intraperitoneally with 11.1-14.8 MBq of ^18^F-FDG (Betaplus Pharma, Brussels, Belgium). A 10 min transmission scan was first obtained in a single mode using a 370 MBq ^137^Cs source for attenuation correction. A 10 min static PET acquisition was then performed after a 60 min resting period. After the correction with attenuation factors obtained from the transmission scan, images were reconstructed using a fully 3D iterative algorithm (3D-RAMLA) in a 128 x 128 x 120 matrix, with a voxel size of 1 mm^3^. After PET acquisition, anesthetized animals were transferred on the same bed from the PET scanner to the CT scanner (x-ray tube voltage: 55 kVp; number of projections: 180; exposure time 1000 ms) for anatomical reference. The CT projections were reconstructed with a voxel size of 0.221 x 0.221 x 0.221 mm^3^. Regions of Interest (ROIs) were delineated on PET images using PMOD software (PMOD™, version 3.403, PMOD technologies Ltd, Zurich, Switzerland). 2D ROIs were established on consecutive transversal slices using a 50% isocontour tool (ROI including the pixel values larger than 50% of the maximum pixel) that semi-automatically defined a 3D Volume of Interest (VOI) around the tissue of interest. To avoid overestimation of the uptake within the VOI, PET/CT fused images where used to discriminate hot pixels coming from the neighboring tissues like urinary bladder. Using the mean uptake within this VOI, the global tracer uptake was assessed in tumors and expressed as percentage of injected dose per gram of tissue (%ID/g).

### Hyperpolarized ^13^C-MRI studies

1-^13^C pyruvic acid (30 μL), containing 15 mM OXO63 and 2.5 mM gadolinium chelate ProHance (Bracco Diagnostics, Milano, Italy), was hyperpolarized at 3.35T and 1.4K using the Hypersense DNP polarizer (Oxford Instruments, Abingdon, UK) according to the manufacturer's instructions. After 60-90 min, the hyperpolarized sample was rapidly dissolved in 4.5 mL of a superheated alkaline buffer that consisted of 50 mM Tris(hydroxymethyl)aminomethane, 75 mM NaOH, and 100 mg/L ethylenediaminetetraacetic acid. The hyperpolarized 1-^13^C pyruvate solution (96 mM) was intravenously injected through a catheter placed in the tail vein of the mouse (12 μL/g body weight). Hyperpolarized ^13^C MRI studies were performed on a 3T scanner (MR Solutions, Guildford, UK) using a home-built ^13^C solenoid leg coil. After the rapid injection of hyperpolarized 1-^13^C pyruvate, spectra were acquired every second for 240 seconds using a single pulse sequence. Data were analyzed in a model free approach using the lactate/pyruvate ratio, calculated from the areas under the curves of the 1-^13^C lactate peak and the 1-^13^C pyruvate peak [15].

### Statistical analysis

Analysis was performed using the GraphPad Prism 7 software. Results are expressed as means value of parameter ± SEM. All statistical tests were two-sided. Paired t-test was used to compare mean changes between groups (baseline vs. post-treatment) for each tumor model, and unpaired t-test was used to compare mean changes between the two tumor models. Results with P < 0.05 (*), <0.01 (**), or <0.001 (***) were considered to be statistically significant.

## SUPPLEMENTARY FIGURES


