# Supplementary material for: Multi-modality imaging to assess metabolic response to dichloroacetate treatment in tumor models
Source: Oncotarget. 2016 Nov 7;7(49):81741–9. doi: 10.18632/oncotarget.13176 (PMC5340254; doi:10.18632/oncotarget.13176)
Supplement: Supplementary file 1 [file oncotarget-07-81741-s001.pdf]

Multi-modality imaging to assess metabolic response to dichloroacetate treatment in tumor models

SUPPLEMENTARY FIGURES

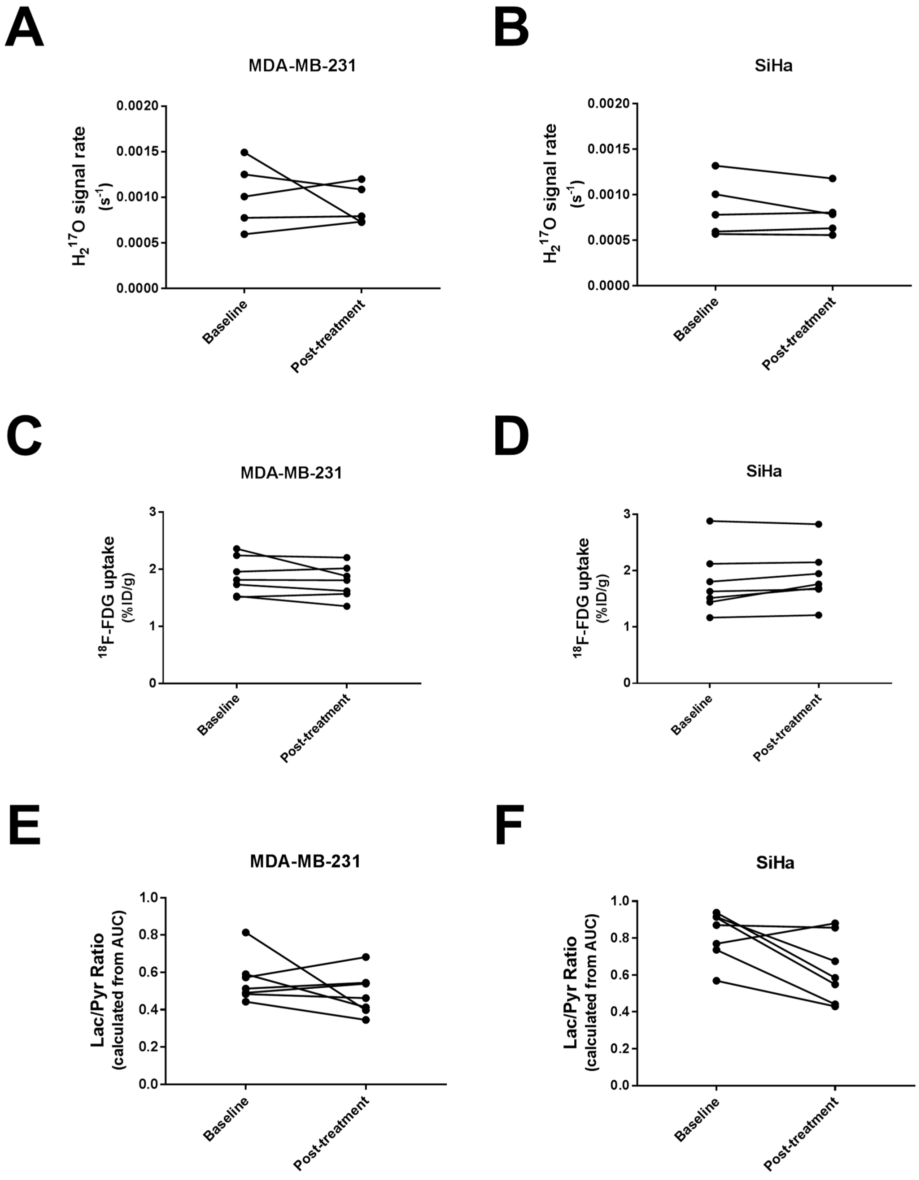

Supplementary Figure S1: Individual tumor responses to DCA treatment in MDA-MB-231 tumors (left side) and SiHa tumors (right side), assessed by $^{17}O_2$ metabolism A-B., ^{18}F-FDG uptake C-D. and pyruvate transformation into lactate E-F.

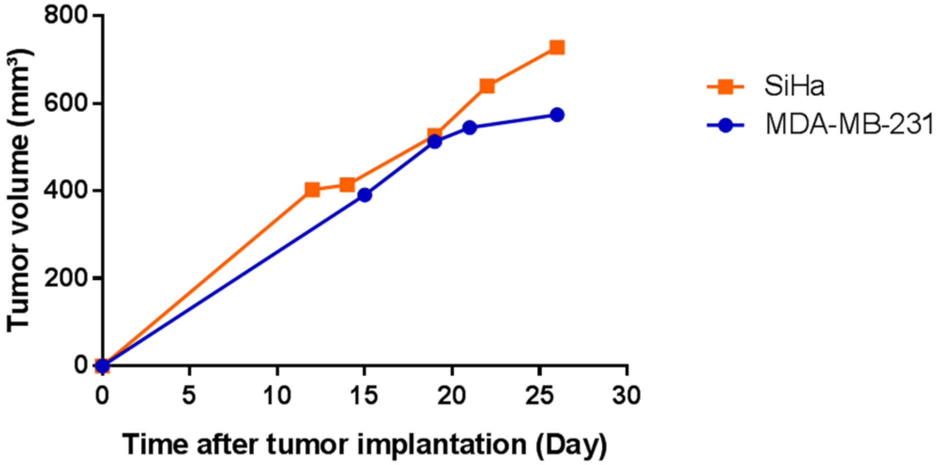

Supplementary Figure S2: Growth curves of MDA-MB-231 and SiHa tumors. Data are expressed as mean values, n = 8 per tumor models.

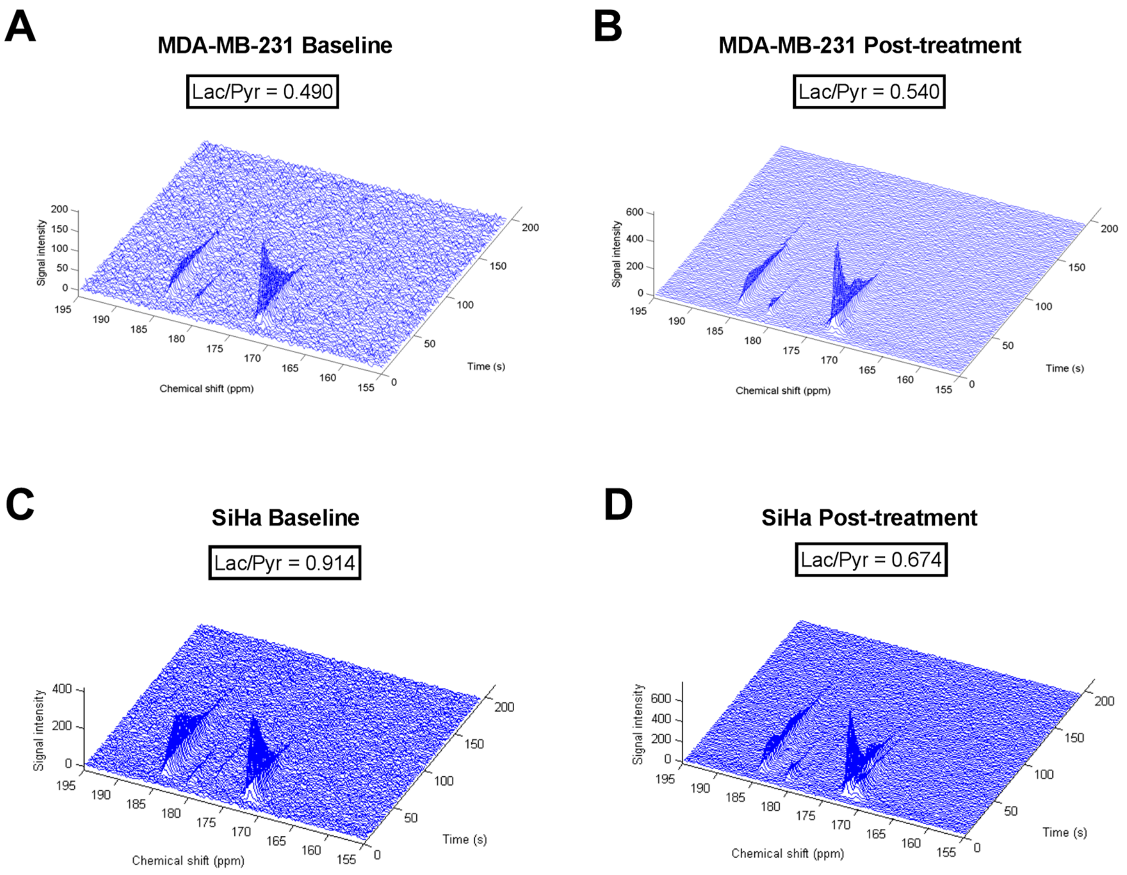

Supplementary Figure S3: Typical ^{13}C-MRS spectra from representative MDA-MB-231 tumors A-B. and SiHa tumors C-D.: pyruvate (173 ppm), lactate (185 ppm) peaks and pyruvate hydrate (181 ppm). Bicarbonate (162 ppm) was not observed before or after treatment, in both tumor models.
